# Patient-Reported Tolerability, Treatment Initiation, and Discontinuation of Postoperative Progestin Therapy After Cesarean Scar Repair in Women Planning Pregnancy

**DOI:** 10.3390/medicina62101996

**Published:** 2026-10-15

**Authors:** Tilman Born, Josef Deglmann, Marie Pohle-Rüffer, Katharina Rathberger, Maurice Kappelmeyer, Angela Köninger, Mehmet Vural

**Affiliations:** Department of Gynecology and Obstetrics, Hospital St. Hedwig, University of Regensburg, Steinmetzstrasse 1-3, 93049 Regensburg, Germany

**Keywords:** cesarean scar disorder, isthmocele, progestin, desogestrel, treatment discontinuation, fertility, patient-reported outcomes

## Abstract

*Background and Objectives:* Cesarean scar disorder (CSDi) may require surgical repair in women with a large niche and thin residual myometrium. At our center, temporary postoperative progestin therapy is recommended to suppress cyclic endometrial proliferation and menstrual activity at the freshly reconstructed scar during early wound healing; whether this improves structural healing is unknown. We evaluated patient-reported treatment initiation, discontinuation, tolerability, bleeding patterns, mood-related symptoms, sexual behavior, and previous hormonal treatment experience in women planning pregnancy. *Materials and Methods:* This retrospective single-center cohort study included 74 women who underwent laparoscopic-assisted cesarean scar repair between 2020 and 2023. Seventy completed questionnaire follow-ups between March and October 2024 (response rate 94.6%). Hormonal exposure was patient-reported and included desogestrel, dienogest, or an unspecified progestin. Analyses were primarily descriptive and used item-specific denominators. *Results:* Among women with valid postoperative treatment information, 43/55 (78.2%) reported progestin use. Very-high motivation or a pregnancy-related motivation response was selected by 43/59 women (72.9%). Premature discontinuation because of adverse effects was reported by 15/61 (24.6%), and prolonged vaginal bleeding >14 days by 15/64 (23.4%). Mean perceived adverse-effect intensity was 3.43 (SD 2.99) on a 10-point scale. Fatigue, irritability, and depressed mood were the most frequently reported mood-related symptoms. Among women comparing the postoperative regimen with previous hormonal preparations, 20/32 (62.5%) reported no relevant difference in tolerability. *Conclusions:* Most women with available treatment information initiated postoperative progestin therapy, and motivation remained substantial despite an active desire for pregnancy. A relevant subgroup reported adverse-effect-related discontinuation or prolonged bleeding. These findings support careful pretreatment counseling and shared decision-making. The effects of postoperative hormonal suppression on anatomical healing and reproductive outcomes remain uncertain.

## 1. Introduction

Cesarean delivery is one of the most frequently performed surgical procedures worldwide. In Germany, the cesarean delivery rate reached 33.0% of hospital births in 2024 [1]. With this high prevalence, the long-term consequences of uterine scar healing have become increasingly relevant. A uterine niche is defined sonographically as an indentation of at least 2 mm at the site of a previous cesarean hysterotomy [2]. When a niche is associated with at least one primary symptom or two secondary symptoms, the clinical condition is defined as cesarean scar disorder (CSDi) [3]. A recent three-year follow-up study after first cesarean delivery reported CSDi in 42.5% of a selected cohort of nonpregnant, nonbreastfeeding women not using hormonal contraception, although the authors emphasized the potential for selection-related overestimation [4].

CSDi may present with postmenstrual spotting, dysmenorrhea, dyspareunia, chronic pelvic pain, abnormal vaginal discharge, subfertility, intrauterine fluid, or technical difficulties during embryo transfer [3]. Niche development is considered multifactorial. Proposed contributors include a low uterine incision involving cervical tissue, incomplete approximation of the myometrium, ischemia or adhesion-related traction, patient-related impairment of wound healing, and uterine retroflexion [5]. Histologic studies have identified endocervical or disorganized lower-segment endometrial mucosa, inclusion cysts, regenerative epithelial changes, and fibroblastic stromal reactions within excised niches [6]. CSDi symptoms may also overlap with endometriosis; in a surgical cohort of women with symptomatic or large uterine niches, extragenital endometriosis was histologically confirmed in nearly half of patients [7].

Treatment should be individualized according to symptoms, residual myometrial thickness (RMT), fertility goals, uterine anatomy, and defect size and configuration. Hysteroscopic resection may reduce bleeding symptoms in selected women with sufficient residual myometrium [8], whereas laparoscopic or combined repair is used for large defects with a thin residual myometrium and can improve scar thickness, postmenstrual spotting, dysmenorrhea, intrauterine fluid, and quality of life [9,10]. High-quality comparative evidence for reproductive outcomes and for adjunctive postoperative measures, however, remain limited [11].

At our center, women undergoing laparoscopic-assisted cesarean scar repair are advised to use temporary postoperative progestin therapy, most commonly desogestrel 75 µg daily. The rationale is to suppress cyclic endometrial proliferation and menstrual activity at the freshly reconstructed scar during the early postoperative period, thereby avoiding cyclic endometrial activity at the repair site while primary wound healing is ongoing. This is a center-specific management concept, not an established strategy to improve myometrial healing; whether menstrual suppression affects structural scar healing or reproductive outcomes remains unknown. Because many women undergo scar repair in the context of future pregnancy, the recommendation temporarily delays pursuit of their reproductive goal and may therefore pose a distinct adherence challenge.

Desogestrel 75 µg is an estrogen-free progestin-only pill that reliably inhibits ovulation. Randomized contraceptive trials have shown good overall acceptability but variable bleeding patterns, including amenorrhea, infrequent bleeding, spotting, and prolonged bleeding [12,13]. The relationship between hormonal contraception and mood is complex. Large observational studies have reported associations with depression-related outcomes [14], whereas individual responses vary and causal interpretation remains limited. Recent prospective contraceptive data indicate that mood-related symptoms may contribute to early discontinuation, including among users of progestin-only methods [15]. Treatment beliefs may also influence adherence, which tends to be stronger when perceived personal necessity outweighs concerns about adverse effects [16]. Sexual acceptability can likewise affect contraceptive satisfaction and continuation over time [17].

This study aimed to characterize patient-reported treatment initiation, duration, premature discontinuation, physical tolerability, mood-related symptoms, bleeding patterns, sexual behavior, and comparisons with previous hormonal preparations in women advised to use temporary postoperative progestin therapy after cesarean scar repair.

## 2. Materials and Methods

This retrospective single-center cohort study with questionnaire-based follow-up was conducted at the Department of Gynecology and Obstetrics, University Hospital St. Hedwig, Regensburg, Germany. Women who underwent cesarean scar repair between January 2020 and December 2023 formed the surgical cohort; questionnaire data were collected from March to October 2024. The analysis focused on patient-reported treatment initiation, treatment course, and tolerability of temporary postoperative progestin therapy in women planning future pregnancy.

The methodological procedures of this study were reviewed and approved by the Ethics Committee of the University of Regensburg, Germany (protocol code 21-2427-101).

**Figure 1:**
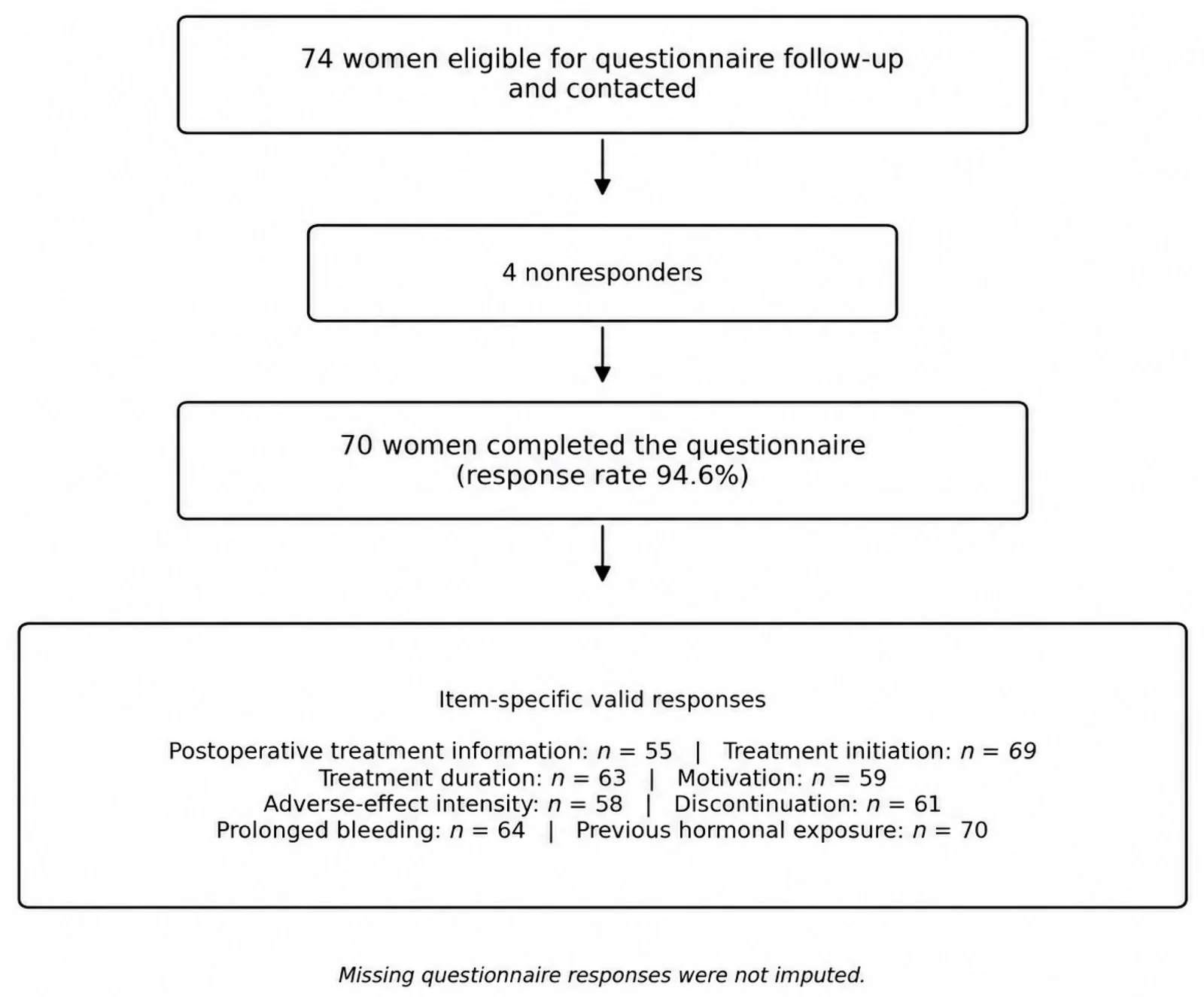
Participant flow and item-specific questionnaire denominators. All 74 eligible women were contacted; 70 completed the questionnaire (94.6% response rate). Item-specific missing responses were not imputed.

All 74 women who had undergone laparoscopic-assisted cesarean scar repair and were eligible for questionnaire follow-up were contacted. Seventy completed the questionnaire, corresponding to an overall response rate of 94.6%, while four did not respond. Baseline clinical data were available for the full eligible cohort (*n* = 74); patient-reported outcomes were analyzed among questionnaire respondents using item-specific denominators. Desogestrel 75 µg daily was the standard postoperative recommendation at our center. Because medication exposure was captured retrospectively by patient report, reported regimens included desogestrel, dienogest, an unspecified progestin, or no postoperative hormonal treatment. The interval between surgery and questionnaire completion ranged from 3 to 58 months, reflecting substantial heterogeneity in the duration of retrospective recall across participants. To explore potential nonresponse bias, available baseline characteristics of respondents and nonrespondents were compared descriptively; with only four nonrespondents, no inferential testing was performed. Participant flow and item-specific questionnaire denominators are summarized in Figure 1.

Women with a uterine niche, defined as an indentation ≥ 2 mm at the previous cesarean scar site on transvaginal sonography (GE Healthcare Austria GmbH & Co OG, Zipf, Austria), were considered for surgical treatment when clinically relevant symptoms were present. These included postmenstrual spotting, dysmenorrhea, dyspareunia, chronic pelvic pain, abnormal vaginal discharge, and unexplained secondary infertility. Symptoms had to have developed or worsened after cesarean delivery and to be clinically attributable to the scar defect. The local surgical eligibility criteria predated publication of the international CSDi definition in 2023 and were applied throughout the study period; selection for surgery was therefore based on sonographic characteristics together with symptom burden. Women undergoing laparoscopic-assisted repair had a residual myometrial thickness < 2 mm. Patients with endometriosis diagnosed before the most recent pregnancy were excluded, as were women with smaller niches and a residual myometrial thickness > 2 mm who were considered suitable for hysteroscopic correction.

Patient-reported experience with postoperative progestin therapy was assessed using a study-specific questionnaire implemented digitally via Qnome^®^ (MaganaMed GmbH, Regensburg, Germany), an online survey platform. Participants received a postal letter containing a personalized QR code and a direct URL to the survey. If no response was recorded within four weeks, patients were contacted by telephone to encourage participation and address possible technical difficulties.

The questionnaire was developed within a broader postoperative follow-up project after cesarean scar repair. The present analysis was restricted to items addressing postoperative progestin use, treatment initiation and duration, premature discontinuation, perceived adverse effects, bleeding and vaginal discharge patterns, mood-related symptoms, sexual behavior, treatment motivation, and previous hormonal treatment experience. Additional items addressing other postoperative research questions were not included in the present analysis. An English translation of the analyzed items is provided as Supplementary Material S1. The questionnaire was study-specific and was not a validated psychometric instrument; it was designed to capture clinically relevant aspects of postoperative treatment experience rather than psychiatric or other diagnostic outcomes. The assessed domains overlap with recognized issues affecting tolerability and continuation of progestin-only and other hormonal contraceptive methods, including bleeding changes, mood-related symptoms, sexual acceptability, and treatment persistence [12,13,14,15,16,17]. Before implementation, the questionnaire was internally pretested by medical colleagues and non-medical administrative staff for comprehensibility, wording, and technical usability. This pretesting did not constitute formal psychometric validation. Women with previous hormonal treatment were also asked to retrospectively compare perceived adverse-effect intensity and overall tolerability of the postoperative regimen with their previous hormonal preparation.

All data were pseudonymized and analyzed using R software (version 4.4.0; R Foundation for Statistical Computing, Vienna, Austria) with the gtsummary package (version 1.7.2; Sjoberg et al., available via CRAN). Analyses were primarily descriptive. Continuous variables are reported as means with standard deviations (SD) and/or medians with interquartile ranges (IQR), and categorical variables as counts and percentages. Because of item-specific nonresponse, denominators vary across outcomes and are reported explicitly. Missing responses were not imputed, and reasons for individual unanswered items were not systematically recorded. The analyses were intended to characterize patient-reported treatment experience rather than establish causal effects of postoperative progestin therapy. For the principal proportions, 95% confidence intervals (CIs) were calculated using the Wilson score method.

The primary analysis pooled all reported postoperative progestin exposures because the study was designed to characterize the real-world postoperative suppression strategy rather than compare individual progestins. To address treatment heterogeneity, an exploratory sensitivity analysis was performed among women with clearly identified desogestrel use (*n* = 32), excluding those who reported dienogest, an unspecified progestin, no hormonal treatment, or missing treatment information. No formal between-drug comparison was undertaken because only three women reported dienogest use. Within the confirmed desogestrel subgroup, two exploratory associations were assessed using Fisher’s exact test: prolonged bleeding (>14 days) versus premature discontinuation because of adverse effects, and previous hormonal contraceptive use versus premature discontinuation.

## 3. Results

Of the 74 eligible women contacted, 70 completed the questionnaire, yielding an overall response rate of 94.6% (95% CI 86.9–97.9%); four women did not respond. Among the 70 respondents, 32 (45.7%) reported postoperative desogestrel use, three (4.3%) reported dienogest use, and eight (11.4%) could not recall the exact preparation. Twelve women (17.1%) reported no postoperative hormonal medication, and 15 (21.4%) provided no information on postoperative pill use. After excluding item-specific missing responses, 43 of 55 women (78.2%; 95% CI 65.6–87.1%) reported postoperative progestin use, whereas 12 of 55 (21.8%) reported no hormonal follow-up treatment.

Baseline clinical characteristics were available for all 74 eligible women. Mean age was 35.9 years (SD 3.9), and median gravidity and parity were both 1.0 (IQR 1.0–2.0). Mean body mass index was 26.5 kg/m^2^ (SD 5.4). Most women had undergone one previous cesarean section (CS) (53/74, 71.6%); 17/74 (23.0%) had undergone two and 4/74 (5.4%) three CS (Table 1). Available baseline characteristics showed no marked differences between the 70 respondents and four nonrespondents. Mean BMI was 26.6 versus 26.2 kg/m^2^; median gravidity, parity, and number of previous cesarean sections were 1.0 versus 1.5 in each comparison. Given the very small nonrespondent group, these comparisons were descriptive only.

Among women with available information on treatment initiation, 45 of 69 (65.2%) started the pill at the beginning of the next menstrual cycle and 13 of 69 (18.8%) a few days after hospital discharge; 11 of 69 (15.9%) reported no pill intake. Treatment duration was one to three months in 28 of 63 women (44.4%) and three to six months in 20 of 63 (31.7%). A very-high motivation response or a pregnancy-related motivation response was selected by 43 of 59 women (72.9%; 95% CI 60.4–82.6%). The most common response was very-high motivation because pill intake was perceived as part of the treatment concept (31/59, 52.5%); a further 12/59 women (20.3%) selected the moderate, pregnancy-related motivation response (Table 2).

Among respondents with available tolerability data, mean perceived adverse-effect intensity during the postoperative treatment period was 3.43 (SD 2.99) on a 10-point numerical rating scale, with a median of 2.0 (IQR 1.0–6.0). Among women with discontinuation data, 46 of 61 (75.4%) reported planned discontinuation, whereas 15 of 61 (24.6%; 95% CI 15.5–36.7%) reported premature discontinuation because of adverse effects.

Prolonged vaginal bleeding lasting more than 14 days was reported by 15 of 64 women (23.4%; 95% CI 14.7–35.1%). Among these women, bleeding lasted less than four weeks in five cases (33.3%), four to eight weeks in three (20.0%), and throughout the reported treatment period in seven (46.7%). Mean perceived bleeding intensity in this subgroup was 6.33 (SD 1.88), with a median of 7.0 (IQR 5.0–8.0). Fatigue, irritability, and depressed mood were the most frequently reported mood-related symptoms; weight gain, irregular bleeding, hair loss, and amenorrhea were the most frequently reported physical symptoms (Table 3).

In an exploratory sensitivity analysis restricted to the 32 women with clearly identified desogestrel use, 7/32 (21.9%; 95% CI 11.0–38.8%) reported premature discontinuation because of adverse effects and 9/32 (28.1%; 95% CI 15.6–45.4%) reported prolonged vaginal bleeding lasting more than 14 days. Among 31 women with available ratings, mean perceived adverse-effect intensity was 3.06 (SD 3.04), with a median of 1.0 (IQR 1.0–5.5). A very-high motivation response or the pregnancy-related motivation response was selected by 25/31 women (80.6%; 95% CI 63.7–90.8%). Overall, the sensitivity analysis yielded a pattern consistent with the primary descriptive findings. Within this subgroup, premature discontinuation occurred in 4/9 women (44.4%) with prolonged bleeding and 3/23 (13.0%) without prolonged bleeding (Fisher’s exact *p* = 0.076). It occurred in 4/21 women (19.0%) with previous hormonal contraceptive use and 3/11 (27.3%) without previous hormonal use (Fisher’s exact *p* = 0.67). These exploratory comparisons were not adjusted for multiple testing and should be interpreted cautiously.

Patient-reported vaginal discharge patterns varied before surgery, during pill intake, and after treatment. Normal, odorless discharge was less frequently reported during pill intake, whereas bloody discharge was more frequently reported during treatment and less frequently thereafter. Viscous and malodorous discharge were uncommon. Discharge occurring on more than 20 days per month was reported by 3 of 70 women (4.3%) before surgery, 5 of 70 (7.1%) during pill intake, and 1 of 70 (1.4%) after treatment. These descriptive data do not allow the effects of surgery, hormonal treatment, and time to be distinguished.

Before surgery, 37 of 70 women (52.9%) had previously used hormonal preparations. Among women with available information on preparation type, combined estrogen–progestin preparations were most common, followed by vaginal rings and progestin-only preparations. Mean adverse-effect intensity with previous hormonal preparations was 3.65 (SD 2.96), with a median of 2.0 (IQR 1.0–6.0), and was numerically similar to that reported during the postoperative regimen. Among 32 women who directly compared the postoperative regimen with previous hormonal treatment, 20 (62.5%) reported no relevant difference in tolerability. Of the 12 who perceived a difference, six considered the postoperative progestin better tolerated and six favored a previous hormonal preparation (Table 4).

## 4. Discussion

This study provides a patient-centered description of treatment initiation, treatment course, tolerability, bleeding patterns, mood-related symptoms, and sexual behavior during temporary postoperative progestin therapy after laparoscopic-assisted cesarean scar repair in women planning pregnancy. Most women with valid medication data reported initiating a progestin regimen, and motivation remained substantial. At the same time, approximately one quarter of women with available discontinuation data reported premature cessation because of adverse effects. The findings therefore characterize the feasibility and patient burden of a center-specific postoperative strategy rather than establish a clinical benefit of hormonal suppression after cesarean scar repair. Recent clinical consensus likewise emphasizes that management of cesarean scar niche should be individualized according to symptoms, residual myometrial thickness, and future fertility plans [18].

Treatment exposure was heterogeneous because a small number of women reported dienogest or could not identify the exact postoperative progestin. Restricting the analysis to women with clearly confirmed desogestrel use, however, yielded a similar pattern of treatment motivation, adverse-effect-related discontinuation, bleeding burden, and perceived adverse-effect intensity. This sensitivity analysis supports the robustness of the descriptive findings but should not be interpreted as a comparison between progestins, as the alternative-treatment groups were too small for meaningful comparative analysis.

The recommendation for temporary hormonal suppression creates an unusual clinical context: women undergo scar repair to improve symptoms and, often, future fertility, yet are asked to defer conception temporarily while taking an ovulation-inhibiting treatment. In this cohort, this tension did not appear to undermine motivation. Very-high motivation because pill intake was perceived as part of the treatment concept was reported by 52.5% of respondents, while a further 20.3% selected the moderate, pregnancy-related motivation response. These responses are consistent with the Necessity–Concerns Framework described by Horne and Weinman [16], in which adherence-related behavior reflects the balance between perceived treatment necessity and concerns about adverse effects. Given the descriptive design and self-reported motivation categories, this interpretation remains hypothesis-generating. The fertility context is nonetheless clinically important: a recent systematic review and meta-analysis found lower live-birth rates after in vitro fertilization (IVF) in women with an isthmocele than in women with a previous cesarean delivery without an isthmocele; outcomes after surgical repair were encouraging, although the quality of evidence for many repair-related outcomes remained low or very low [19]. Even a temporary delay in attempts to conceive may therefore be highly salient to this population.

Bleeding-related tolerability emerged as an important component of treatment burden. Prolonged bleeding lasting more than 14 days was reported by 23.4% of respondents to this item, and perceived bleeding intensity in this subgroup was high. Irregular and unpredictable bleeding is a well-recognized feature of progestin-only contraception [12,13] and may be particularly burdensome after surgery in women expecting symptom improvement and wishing to proceed toward pregnancy. Pretreatment counseling should therefore address the possibility of spotting or prolonged bleeding, together with the temporary nature and rationale of the proposed regimen. Although mean overall adverse-effect intensity was modest, individual experiences varied substantially, as reflected by the proportion reporting premature discontinuation because of adverse effects. Current Faculty of Sexual and Reproductive Healthcare (FSRH) guidance likewise notes that bleeding patterns during desogestrel progestogen-only pill use are unpredictable and may contribute to discontinuation [20]. In the prospective real-world CONTENT study, 25.8% of women using a progestogen-only pill discontinued their prescribed treatment during follow-up [21]. This rate is not directly comparable with the 24.6% adverse-effect-related discontinuation observed in our postoperative cohort because treatment indication, follow-up duration, and definitions differed, but it illustrates that discontinuation is also common in routine progestogen-only pill use. Earlier prospective data similarly found that 46% of women who discontinued a newly prescribed oral contraceptive attributed discontinuation to side effects [22]. Taken together, these findings underscore the importance of anticipatory counseling about bleeding changes and other potential adverse effects before temporary postoperative progestin therapy is initiated.

Mood-related symptoms were frequently reported during treatment, most commonly fatigue, irritability, and depressed mood. These observations align with the broader literature showing heterogeneous associations between hormonal contraception, mood-related symptoms, and method discontinuation [14,15]. The questionnaire items, however, capture patient-reported symptoms rather than validated psychological outcomes. No validated psychometric instruments or pretreatment mood assessments were used, and there was no untreated control group; causal attribution to progestin therapy is therefore not possible. Changes in sexual behavior were reported by 34.9% of respondents to this item, predominantly as reduced sexual activity. In this postoperative setting, sexual behavior is likely multifactorial and may be influenced by recovery, pain, medical advice regarding sexual activity, fertility-related concerns, relationship factors, and hormonal treatment itself [17]. Among women able to compare regimens, 62.5% reported no relevant difference in tolerability between the postoperative treatment and previously used hormonal preparations, indicating that the postoperative regimen was not uniformly perceived as less tolerable than prior hormonal exposure. Current guidance and recent systematic review evidence likewise support a cautious interpretation of mood-related complaints: available data do not demonstrate a clear general causal association between progestin-only contraception and depression, and findings across oral contraceptive studies remain heterogeneous [20,23].

Vaginal discharge patterns also varied across the perioperative periods, with bloody discharge reported more often during pill intake and less often after treatment. These observations should be interpreted descriptively because the effects of surgery, postoperative recovery, hormonal exposure, and time cannot be separated in the absence of an untreated comparison group. More broadly, evidence for adjunctive medical therapy after cesarean scar repair remains limited [11]. At our center, the rationale for temporary postoperative suppression is to avoid cyclic endometrial proliferation and menstrual activity at the freshly reconstructed scar while primary wound healing is ongoing, not to directly enhance myometrial healing. Whether this strategy influences scar remodeling, residual myometrial thickness, niche recurrence, or subsequent reproductive outcomes is unknown and was not assessed here. Bleeding changes during active treatment may likewise reflect the pharmacologic effect of menstrual suppression rather than structural effects of scar repair. The present findings therefore support a pragmatic counseling approach rather than any claim of treatment efficacy: postoperative progestin therapy appears acceptable to many motivated patients, but bleeding disturbances, mood-related symptoms, and other adverse effects may limit persistence in a relevant subgroup.

## 5. Strengths and Limitations

A particular strength of this study is its focus on a clinically relevant but understudied aspect of care after cesarean scar repair: how women planning future pregnancy experience a temporary course of postoperative hormonal suppression. The study-specific digital questionnaire captured several patient-centered domains, including treatment initiation and persistence, bleeding patterns, perceived adverse effects, sexual behavior, motivation, and comparison with previous hormonal treatment experience. This provides a broader view of treatment acceptability than medication continuation alone.

Several limitations should be considered when interpreting these findings. The relatively small sample and single-center design limit precision and generalizability, and the retrospective design is inherently susceptible to recall bias. Surgery and postoperative treatment occurred between 2020 and 2023, whereas questionnaire data were collected from March to October 2024. The interval between surgery and questionnaire completion ranged from 3 to 58 months and therefore varied substantially between participants. This heterogeneity in recall duration should be considered when interpreting the retrospective questionnaire data. Recall bias may consequently have affected reported treatment duration, bleeding patterns, perceived adverse effects, and reasons for discontinuation. Although the overall questionnaire response rate was high (94.6%), item-specific nonresponse resulted in smaller denominators for several outcomes and may have introduced additional response bias. Missing responses were not imputed.

Medication exposure and outcomes were patient-reported, and the exact postoperative preparation was not recalled by all participants. The questionnaire was study-specific and had not undergone formal psychometric validation, although it was internally pretested for comprehensibility and usability. Mood-related symptoms, motivation, perceived adverse-effect intensity, and sexual behavior should therefore be interpreted as descriptive patient-reported outcomes rather than validated psychological or behavioral measures. Prospective pretreatment baselines were not available for these domains. Women with previous hormonal exposure did provide a retrospective within-person comparison of overall adverse-effect intensity and tolerability, but this does not replace prospective baseline assessment. In addition, changes in sexual behavior and other reported symptoms cannot be attributed specifically to progestin exposure, as postoperative recovery, pain, medical recommendations, fertility-related concerns, and other contextual factors may have contributed.

The absence of an expectantly managed control group also precludes conclusions about whether postoperative hormonal suppression improves scar healing or other clinical outcomes. Reproductive outcomes, including pregnancy, time to conception, miscarriage, and live birth, were outside the predefined scope of this patient-reported adherence and tolerability study and were not systematically or longitudinally assessed. Although the broader postoperative questionnaire contained exploratory fertility-related items, follow-up was not standardized and these data were not designed to establish reproductive outcome rates. Such outcomes should be addressed in studies specifically designed for that purpose.

## 6. Conclusions

Temporary postoperative progestin therapy was initiated by most women with available treatment data, and motivation remained substantial despite the competing goal of pregnancy. Nevertheless, approximately one quarter of women with available discontinuation data reported premature cessation because of adverse effects, and prolonged bleeding affected a relevant subgroup. Mood-related symptoms and changes in sexual behavior were also reported but cannot be causally attributed to progestin therapy in this retrospective study. These findings support transparent pretreatment counseling and shared decision-making about treatment duration, expected bleeding changes, and potential treatment burden. Prospective controlled studies are needed to determine whether this center-specific strategy influences anatomical healing or reproductive outcomes after cesarean scar repair.

## Figures and Tables

**Table 1:** Demographic and baseline characteristics of the study cohort (*n* = 74).

**Characteristic**	**Mean (SD) or *n* (%)**	**Median (IQR)**
Age, years	35.9 (3.9)	36.5 (33.0–39.0)
Gravidity, *n*	-	1.0 (1.0–2.0)
Parity, *n*	-	1.0 (1.0–2.0)
Height, cm	165 (7)	166 (160–170)
Weight, kg	73 (16)	71 (61–81)
Body mass index, kg/m^2^	26.5 (5.4)	25.1 (22.9–29.4)
**Number of previous cesarean sections**		
1	53 (71.6%)	
2	17 (23.0%)	
3	4 (5.4%)	

Values are presented as mean (SD), median (IQR), or *n* (%). Percentages refer to the overall cohort unless otherwise indicated.

**Table 2:** Postoperative progestin regimen, treatment initiation, duration, motivation, and sexual behavior.

**Characteristic**	***n***	**Result**
**Postoperative preparation reported**		
Desogestrel	70	32 (45.7%)
Dienogest	70	3 (4.3%)
Preparation name unknown	70	8 (11.4%)
No hormonal follow-up treatment	70	12 (17.1%)
No information on pill use	70	15 (21.4%)
Any postoperative progestin use among valid responses	55	43 (78.2%)
**Timing of treatment initiation**		
With beginning of the next menstrual cycle	69	45 (65.2%)
A few days after hospital discharge	69	13 (18.8%)
No pill intake	69	11 (15.9%)
**Duration of pill intake**		
<1 month	63	8 (12.7%)
1–3 months	63	28 (44.4%)
3–6 months	63	20 (31.7%)
>6 months	63	7 (11.1%)
**Motivation for pill intake**		
Very high: perceived as part of treatment	59	31 (52.5%)
Moderate: related to desire for future pregnancy	59	12 (20.3%)
Moderate: concerns about adverse effects	59	3 (5.1%)
Low: previous negative hormonal experience	59	13 (22.0%)
Very-high motivation or pregnancy-related motivation	59	43 (72.9%)
**Sexual behavior during pill intake**		
No change in sexual behavior	63	41 (65.1%)
Any change in sexual behavior	63	22 (34.9%)
Reduced sexual activity	21	17 (81.0%)
New-onset dyspareunia	21	3 (14.3%)
Increased sexual activity	21	1 (4.8%)

Values are *n* (%). Denominators vary because of item-specific nonresponse and reflect the available responses to each questionnaire item. Dienogest is shown separately because three women reported its use instead of desogestrel.

**Table 3:** Patient-reported adverse effects and bleeding-related tolerability during the postoperative treatment period.

** Characteristic **	***n***	**Mean (SD) or *n* (%)**	**Median (IQR)**
Perceived adverse-effect intensity during the treatment period, NRS 1–10	58	3.43 (2.99)	2.0 (1.0–6.0)
**Reason for treatment discontinuation**			
Planned discontinuation	61	46 (75.4%)	
Unplanned discontinuation because of adverse effects	61	15 (24.6%)	
**Prolonged vaginal bleeding**			
Vaginal bleeding > 14 days during treatment	64	15 (23.4%)	
Duration < 4 weeks	15	5 (33.3%)	
Duration 4–8 weeks	15	3 (20.0%)	
Duration throughout entire treatment period	15	7 (46.7%)	
Perceived bleeding intensity, NRS 1–10	15	6.33 (1.88)	7.0 (5.0–8.0)
**Bleeding pattern reported**			
Continuous spotting	70	6 (8.6%)	
Intermittent spotting	70	4 (5.7%)	
Prolonged heavy bleeding	70	5 (7.1%)	
Spotting/intermenstrual bleeding	70	3 (4.3%)	
Mucus admixture	70	2 (2.9%)	
No bleeding pattern noticed	70	1 (1.4%)	
**Mood-related symptoms reported during the treatment period**			
Fatigue	70	21 (30.0%)	
Irritability	70	19 (27.1%)	
Depressed mood	70	16 (22.9%)	
Sleep disturbances	70	8 (11.4%)	
**Physical symptoms reported during the treatment period**			
Weight gain	70	18 (25.7%)	
Irregular bleeding	70	15 (21.4%)	
Hair loss	70	14 (20.0%)	
Amenorrhea	70	12 (17.1%)	
Breast pain	70	7 (10.0%)	
Acne	70	5 (7.1%)	
Gastrointestinal complaints	70	4 (5.7%)	
Nausea/vomiting	70	4 (5.7%)	

NRS, numerical rating scale. Mood-related and physical symptom categories were not mutually exclusive. Denominators vary by questionnaire item. For symptom items reported with *n* = 70, percentages refer to the respondent cohort and should be interpreted as reporting frequencies rather than treatment-specific incidence.

**Table 4:** Previous hormonal treatment experience and comparison with the postoperative progestin regimen.

**Characteristic**	***n***	**Result**
**Previous hormonal preparation before treatment**		
No	70	33 (47.1%)
Yes	70	37 (52.9%)
**Type of previous hormonal preparation**		
Combined estrogen–progestin preparation	32	15 (46.9%)
Progestin-only preparation	32	6 (18.8%)
Vaginal ring	32	7 (21.9%)
Contraceptive patch	32	1 (3.1%)
Depot preparation	32	1 (3.1%)
Hormone implant	32	0 (0.0%)
Intrauterine hormone system	32	2 (6.3%)
**Adverse effects and comparative tolerability**		
Perceived adverse-effect intensity with previous hormonal preparations, NRS 1–10	37	3.65 (2.96); median 2.0 (IQR 1.0–6.0)
No relevant difference between postoperative and previous preparation	32	20 (62.5%)
Any perceived difference between postoperative and previous preparation	32	12 (37.5%)
Current postoperative progestin better tolerated among those reporting a difference	12	6 (50.0%)
Previous hormonal preparation better tolerated among those reporting a difference	12	6 (50.0%)
**Indications for previous hormonal treatment, multiple responses possible**		
Contraception	70	33 (47.1%)
Dysmenorrhea/period pain	70	6 (8.6%)
Irregular cycle	70	5 (7.1%)
Known or suspected endometriosis	70	4 (5.7%)
Acne/skin symptoms	70	3 (4.3%)
Hair loss	70	2 (2.9%)

NRS, numerical rating scale. Values are *n* (%) unless otherwise indicated.

## Data Availability

The data presented in this study are available on reasonable request from the corresponding author, subject to applicable privacy and ethical restrictions.

## Supplementary Materials

The following supporting information can be downloaded at: https://www.mdpi.com/article/10.3390/medicina62101996/s1, Supplementary Material S1 provides an English translation of the questionnaire items analyzed in the present study. The original questionnaire was administered in German and was developed as part of a broader postoperative follow-up project after cesarean scar repair.

## Abbreviations

BMI	Body mass index
CS	Cesarean section
CSDi	Cesarean scar disorder
FSRH	Faculty of Sexual and Reproductive Healthcare
IVF	In vitro fertilization
IQR	Interquartile range
NRS	Numerical rating scale
RMT	Residual myometrial thickness
SD	Standard deviation
