# Supplementary material for: Patient-Reported Tolerability, Treatment Initiation, and Discontinuation of Postoperative Progestin Therapy After Cesarean Scar Repair in Women Planning Pregnancy
Source: Medicina (Kaunas). 2026 Oct 15;62(10):1996. doi: 10.3390/medicina62101996 (PMC13707190; doi:10.3390/medicina62101996)
Supplement: Supplementary file 1 [file medicina-62-01996-s001.zip › medicina-4554338-supplementary.pdf]

Supplementary Material S1

English Translation of Questionnaire Items Analyzed in the Present Study

Note. The original questionnaire was administered in German and was developed as part of a broader postoperative follow-up project after cesarean scar repair. Only the items relevant to postoperative progestin treatment experience and analyzed in the present manuscript are reproduced here. The questionnaire was study-specific and was not formally validated. Before implementation, it was internally pretested by medical colleagues and non-medical administrative staff for comprehensibility, wording, and technical usability. Wording and response categories are translated from the original German questionnaire.

1. Which pill did you take postoperatively?

- Free-text field

2. When did you start taking the pill after surgery?

- A few days after hospital discharge
- At the beginning of the next menstrual cycle
- No pill taken

3. How would you rate the intensity of the adverse effects you experienced while taking the preparation? (1 = no noticeable adverse effects; 10 = adverse effects intolerable/treatment discontinuation)

- Numerical rating scale: 1-10

4. How long did you take the pill?

- <1 month
- 1-3 months
- 3-6 months
- More than 6 months

5. What was the reason for discontinuing the pill?

- Planned discontinuation after 3 or 6 months
- Unplanned discontinuation because of adverse effects

6. Did you experience vaginal bleeding lasting longer than 14 days while taking the pill?

- No
- Yes

7. How long did this vaginal bleeding last?

- <4 weeks
- Between 4 and 8 weeks
- Throughout the entire period of pill use

8. Did you notice a bleeding pattern?

- Continuous spotting
- Spotting with intermittent bleeding-free intervals
- Continuous menstrual-level bleeding
- Intermenstrual bleeding
- Mucus admixture

- No

9. How would you rate the intensity of your bleeding on a scale from 1 to 10, where 1 indicates hardly any bleeding and 10 the strongest imaginable bleeding?

- Numerical rating scale: 1-10

10. Did you notice vaginal discharge? Please select all applicable categories.

- Time periods: before surgery; during pill use; currently
- Viscous
- Malodorous
- Normal (odorless, milky-white)
- Whitish, lumpy/crumbly
- Bloody
- Yellowish/greenish

11. On how many days per month did/does the discharge occur?

- Time periods: before surgery; during pill use; currently
- <5 days/month
- 5-20 days/month
- >20 days/month

12. Did your sexual behavior change during the period of pill use?

- No
- Yes

13. If yes, how did it change?

- Less sexual activity
- More sexual activity
- New-onset pain during sexual intercourse

14. If you experienced any of the following symptoms after surgery and during hormonal treatment, please select all that apply:

- Sleep problems
- Irritability
- Depressed mood
- Fatigue/lack of energy

15. Did you additionally experience any of the following adverse effects?

- Gastrointestinal complaints
- Nausea/vomiting
- Acne
- Breast pain
- Irregular bleeding
- Amenorrhea
- Weight gain
- Hair loss

16. Describe your motivation to take the oral contraceptive pill after surgery.

- Very high motivation because I knew this was part of the treatment
- Moderate motivation because I wanted/want to have children
- Moderate motivation because I was concerned about possible adverse effects

- Low motivation because I had always tolerated pills poorly in the past or know women who experienced severe adverse effects

17. Before surgery, had you previously taken a pill or another hormonal contraceptive?

- No
- Yes

18. If yes, which type of preparation?

- Combined estrogen-progestin preparation
- Progestin-only preparation (micro-pill)
- Vaginal ring
- Contraceptive patch
- Depot preparation ("three-month injection")
- Hormonal implant/subdermal rod
- Levonorgestrel-releasing intrauterine system

19. What was the trade name of the previous preparation (if known)?

- Free-text field

20. What was the reason for taking the previous hormonal treatment? (Multiple responses possible)

- Contraception
- Period pain/dysmenorrhea
- Irregular cycle
- Known or suspected endometriosis
- Acne/skin symptoms
- Hair loss

21. How would you rate the intensity of adverse effects experienced with your previous preparation? (1 = no noticeable adverse effects; 10 = adverse effects intolerable/treatment discontinuation)

- Numerical rating scale: 1-10

22. Was there a difference in adverse-effect intensity between the preparation taken after surgery and preparations used in the past?

- No
- Yes

23. How did this difference manifest?

- I tolerated the current pill better
- I tolerated previous pills/hormonal preparations better
